# Ethyl 2-chloro-[2-(4-chloro­phen­yl)hydrazin-1-yl­idene]acetate

**DOI:** 10.1107/S1600536810032599

**Published:** 2010-08-21

**Authors:** Abdullah M. Asiri, Mohie E. M. Zayed, Seik Weng Ng

**Affiliations:** a Chemistry Department, Faculty of Science, King Abdul Aziz University, PO Box 80203, Jeddah 21589, Saudi Arabia; b Department of Chemistry, University of Malaya, 50603 Kuala Lumpur, Malaysia

## Abstract

The title compound, C_10_H_10_Cl_2_N_2_O_2_, features a planar C_ar_—N(H)—N=C(Cl) unit [torsion angle = 5.5 (4)°] whose benzene substituent is coplanar with it [dihedral angle = 4.7 (4)°]; this unit is slightly twisted with respect to the carboxyl –CO_2_ fragment [dihedral angle = 2.2 (52)°]. The amino group acts as a hydrogen-bond donor to the carbonyl O atom of an adjacent mol­ecule; the hydrogen bond generates a helical polymer that runs along the *b* axis of the monoclinic unit cell.

## Related literature

For a review of the reactions of hydrazonyl halides with heterocyclic thio­nes for heteroannulation, the synthesis of spiro­heterocycles and heterocyclic ring formation, see: Shawali & Farghaly (2008 ▶). For related structures, see: Xu (2006 ▶); Yin *et al.* (2006 ▶).
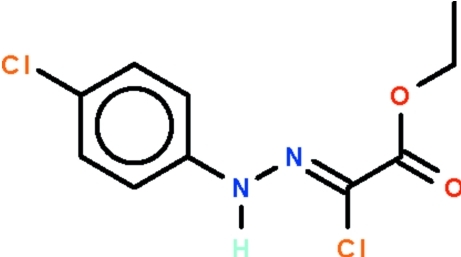


## Experimental

### 

#### Crystal data

C_10_H_10_Cl_2_N_2_O_2_
*M* *_r_* = 261.10
Monoclinic, 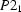

*a* = 4.4611 (7) Å
*b* = 9.4546 (14) Å
*c* = 13.464 (2) Å
β = 91.642 (2)°
*V* = 567.65 (15) Å^3^
*Z* = 2
Mo *K*α radiation
μ = 0.56 mm^−1^
*T* = 100 K
0.35 × 0.10 × 0.05 mm

#### Data collection

Bruker SMART APEX diffractometer
Absorption correction: multi-scan (*SADABS*; Sheldrick, 1996 ▶) *T*_min_ = 0.829, *T*_max_ = 0.973
5298 measured reflections
2518 independent reflections
2191 reflections with *I* > 2σ(*I*)
*R*_int_ = 0.073

#### Refinement

*R*[*F* ^2^ > 2σ(*F* ^2^)] = 0.072
*wR*(*F* ^2^) = 0.188
*S* = 1.03
2518 reflections
145 parameters
1 restraint
H-atom parameters constrained
Δρ_max_ = 0.59 e Å^−3^
Δρ_min_ = −0.34 e Å^−3^
Absolute structure: Flack (1983 ▶), 1123 Friedel pairs
Flack parameter: 0.03 (14)

### 

Data collection: *APEX2* (Bruker, 2009 ▶); cell refinement: *SAINT* (Bruker, 2009 ▶); data reduction: *SAINT*; program(s) used to solve structure: *SHELXS97* (Sheldrick, 2008 ▶); program(s) used to refine structure: *SHELXL97* (Sheldrick, 2008 ▶); molecular graphics: *X-SEED* (Barbour, 2001 ▶); software used to prepare material for publication: *publCIF* (Westrip, 2010 ▶).

## Supplementary Material

Crystal structure: contains datablocks global, I. DOI: 10.1107/S1600536810032599/nk2055sup1.cif

Structure factors: contains datablocks I. DOI: 10.1107/S1600536810032599/nk2055Isup2.hkl

Additional supplementary materials:  crystallographic information; 3D view; checkCIF report

## Figures and Tables

**Table 1:** Hydrogen-bond geometry (Å, °)

*D*—H⋯*A*	*D*—H	H⋯*A*	*D*⋯*A*	*D*—H⋯*A*
N1—H1⋯O1^i^	0.86	2.20	3.009 (5)	156

Symmetry code: (i) 
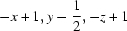
.

**Fig. 1.**
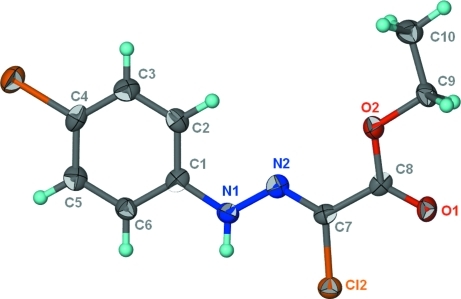
Displacement ellipsoid plot of C10H10Cl2N2O2 at the 70% probability level; hydrogen atoms are drawn as spheres of arbitrary radius.

**Fig. 2.**
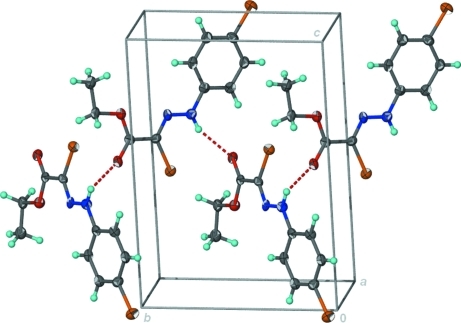
The hydrogen bonded chain structure (red dashed lines) forming a helical chain that runs along the b axis.

## supplementary crystallographic information

### Comment

Ethyl 2-chloro(phenylhydrazono)acetate belongs to the class of of hydrazonyl halides that undergo heteroannulation, and are used for the synthesis of spiroheterocycles and other heterocyclic compounds. The utility in some aspects of heterocyclic chemistry has recently been reviewed (Shawali & Farghaly (2008). The central structural feature is an planar C_ary_l–NH–N═C unit, as noted in the crystal structures of other substituted derivatives (Xu, 2006; Yin *et al.*, 2006). The chlorine-substituted compound (Scheme I) shows this characteristic linkage, whose torsion angle is 5.5?(41) °. The carbon-nitrogen double bond is of a *Z*-configuration (Fig. 1). Such a configuration allows the amino site to form a hydrogen bond to the double-bond carbonyl oxygen atom of an adjacent molecule, this hydrogen bond giving rise to a helical chain that runs along the *b* axis of the unit cell (Fig. 2).

### Experimental

The synthesis works with either 3-chloropentane-2,4-dione or ethyl 2-chloro-3-oxobutanoate. To a solution of either 3-chloropentane-2,4-dione (1.34 g, 10 mmol) or ethyl 2-chloro-3-oxobutanoate (1.64 g, 10 mmol) in ethanol (100 ml) was added sodium acetate trihydrate (1.3 g, 10 mmol). The mixture was chilled to 273 K. To the mixture was added a cold solution of *p*-chlorobenzenediazonium chloride, prepared by diazotizing *p*-chloroaniline (1.20 g, 10 mmol) dissolved in 6*M* hydrochloricacid (6 ml) with a solution of sodium nitrite (0.7 g, 10 mmol) dissolved in water (10 ml). The diazonium salt was added over a period of 20 min. The reaction mixture was stirred for another 15 min. and then left for 3 h in a refrigerator. The resulting solid was collected and washed with water. The crude product was recrystallized from ethanol to give the hydrazone in 85% yield; m.p. 428–431 K.

### Refinement

Carbon-bound H-atoms were placed in calculated positions [C–H 0.95 to 0.99 Å, *U*(H) 1.2 to 1.5*U*_eq_(C)] and were included in the refinement in the riding model approximation. The amino H-atom was similarly positioned [N–H 0.86 Å, *U*(H) 1.2*U*_eq_(N)]. The absolute structure parameter (Flack, 1983) was determined from 1123 Friedel pairs.

### Crystal data

**Table:**

C_10_H_10_Cl_2_N_2_O_2_	*F*(000) = 268
*M**_r_* = 261.10	*D*_x_ = 1.528 Mg m^−^^3^
Monoclinic, *P*2_1_	Mo *K*α radiation, λ = 0.71073 Å
Hall symbol: P 2yb	Cell parameters from 1574 reflections
*a* = 4.4611 (7) Å	θ = 2.6–27.2°
*b* = 9.4546 (14) Å	µ = 0.56 mm^−^^1^
*c* = 13.464 (2) Å	*T* = 100 K
β = 91.642 (2)°	Prism, colourless
*V* = 567.65 (15) Å^3^	0.35 × 0.10 × 0.05 mm
*Z* = 2	

### Data collection

**Table:**

Bruker SMART APEX diffractometer	2518 independent reflections
Radiation source: fine-focus sealed tube	2191 reflections with *I* > 2σ(*I*)
graphite	*R*_int_ = 0.073
ω scans	θ_max_ = 27.5°, θ_min_ = 2.6°
Absorption correction: multi-scan (*SADABS*; Sheldrick, 1996)	*h* = −5→5
*T*_min_ = 0.829, *T*_max_ = 0.973	*k* = −12→11
5298 measured reflections	*l* = −17→17

### Refinement

**Table:**

Refinement on *F*^2^	Secondary atom site location: difference Fourier map
Least-squares matrix: full	Hydrogen site location: inferred from neighbouring sites
*R*[*F*^2^ > 2σ(*F*^2^)] = 0.072	H-atom parameters constrained
*wR*(*F*^2^) = 0.188	*w* = 1/[σ^2^(*F*_o_^2^) + (0.1216*P*)^2^ + 0.1253*P*] where *P* = (*F*_o_^2^ + 2*F*_c_^2^)/3
*S* = 1.03	(Δ/σ)_max_ = 0.001
2518 reflections	Δρ_max_ = 0.59 e Å^−^^3^
145 parameters	Δρ_min_ = −0.34 e Å^−^^3^
1 restraint	Absolute structure: Flack (1983), 1123 Friedel pairs
Primary atom site location: structure-invariant direct methods	Flack parameter: 0.03 (14)

### Fractional atomic coordinates and isotropic or equivalent isotropic displacement parameters (Å^2^)

**Table:**

	*x*	*y*	*z*	*U*_iso_*/*U*_eq_	
Cl1	1.4745 (3)	0.50000 (14)	0.97204 (9)	0.0301 (4)	
Cl2	0.4463 (3)	0.85685 (12)	0.46296 (8)	0.0226 (3)	
N1	0.8095 (9)	0.7704 (5)	0.6374 (3)	0.0203 (8)	
H1	0.8376	0.7407	0.5780	0.024*	
N2	0.6137 (9)	0.8722 (4)	0.6540 (3)	0.0194 (8)	
O1	0.0631 (7)	1.0838 (4)	0.5375 (2)	0.0222 (7)	
O2	0.2281 (8)	1.0713 (4)	0.6974 (2)	0.0223 (7)	
C1	0.9702 (11)	0.7120 (5)	0.7183 (4)	0.0193 (10)	
C2	0.9430 (11)	0.7619 (5)	0.8146 (4)	0.0212 (10)	
H2A	0.8163	0.8403	0.8270	0.025*	
C3	1.0993 (12)	0.6981 (5)	0.8922 (4)	0.0240 (10)	
H3	1.0804	0.7324	0.9581	0.029*	
C4	1.2829 (11)	0.5844 (6)	0.8740 (4)	0.0229 (10)	
C5	1.3191 (11)	0.5353 (5)	0.7776 (4)	0.0225 (10)	
H5	1.4509	0.4587	0.7654	0.027*	
C6	1.1616 (10)	0.5990 (5)	0.7000 (4)	0.0207 (10)	
H6	1.1836	0.5657	0.6340	0.025*	
C7	0.4460 (10)	0.9208 (5)	0.5837 (3)	0.0176 (9)	
C8	0.2257 (10)	1.0339 (5)	0.6023 (3)	0.0183 (9)	
C9	0.0103 (11)	1.1798 (5)	0.7225 (4)	0.0223 (10)	
H9A	−0.1945	1.1501	0.7012	0.027*	
H9B	0.0575	1.2700	0.6891	0.027*	
C10	0.0316 (13)	1.1969 (6)	0.8335 (4)	0.0282 (11)	
H10A	−0.1110	1.2694	0.8541	0.042*	
H10B	0.2356	1.2258	0.8534	0.042*	
H10C	−0.0159	1.1068	0.8654	0.042*	

### Atomic displacement parameters (Å^2^)

**Table:**

	*U*^11^	*U*^22^	*U*^33^	*U*^12^	*U*^13^	*U*^23^
Cl1	0.0349 (7)	0.0288 (7)	0.0262 (6)	0.0030 (5)	−0.0055 (5)	0.0046 (5)
Cl2	0.0251 (6)	0.0219 (6)	0.0209 (5)	−0.0002 (5)	0.0003 (4)	−0.0019 (5)
N1	0.0213 (19)	0.021 (2)	0.0193 (19)	0.0011 (17)	0.0023 (15)	−0.0014 (15)
N2	0.0244 (19)	0.0112 (18)	0.0225 (19)	−0.0033 (16)	0.0012 (14)	0.0034 (16)
O1	0.0253 (18)	0.0156 (17)	0.0253 (17)	0.0035 (14)	−0.0032 (13)	0.0017 (13)
O2	0.0252 (18)	0.0187 (17)	0.0230 (17)	0.0050 (14)	−0.0007 (13)	−0.0012 (13)
C1	0.019 (2)	0.018 (3)	0.022 (2)	−0.0082 (19)	−0.0025 (16)	0.0008 (19)
C2	0.022 (2)	0.015 (2)	0.027 (3)	−0.0014 (19)	0.0028 (19)	−0.0011 (18)
C3	0.029 (3)	0.019 (3)	0.023 (2)	−0.004 (2)	0.0002 (19)	−0.0018 (19)
C4	0.020 (2)	0.023 (2)	0.025 (2)	−0.0036 (19)	−0.0055 (18)	0.0060 (19)
C5	0.025 (2)	0.015 (2)	0.027 (2)	−0.0026 (19)	−0.0011 (18)	−0.0014 (18)
C6	0.017 (2)	0.022 (3)	0.023 (2)	0.0001 (19)	0.0012 (17)	−0.0020 (19)
C7	0.018 (2)	0.018 (2)	0.017 (2)	−0.0054 (18)	−0.0012 (16)	−0.0019 (17)
C8	0.019 (2)	0.016 (2)	0.020 (2)	−0.0081 (18)	−0.0016 (16)	0.0038 (17)
C9	0.025 (3)	0.014 (2)	0.028 (2)	0.003 (2)	0.000 (2)	−0.0028 (18)
C10	0.038 (3)	0.021 (3)	0.026 (2)	0.001 (2)	0.002 (2)	−0.003 (2)

### Geometric parameters (Å, °)

**Table:**

Cl1—C4	1.745 (5)	C3—C4	1.378 (7)
Cl2—C7	1.735 (5)	C3—H3	0.9500
N1—N2	1.323 (6)	C4—C5	1.392 (7)
N1—C1	1.400 (6)	C5—C6	1.381 (7)
N1—H1	0.8600	C5—H5	0.9500
N2—C7	1.275 (6)	C6—H6	0.9500
O1—C8	1.214 (6)	C7—C8	1.478 (7)
O2—C8	1.328 (6)	C9—C10	1.504 (7)
O2—C9	1.459 (6)	C9—H9A	0.9900
C1—C2	1.388 (7)	C9—H9B	0.9900
C1—C6	1.394 (7)	C10—H10A	0.9800
C2—C3	1.379 (7)	C10—H10B	0.9800
C2—H2A	0.9500	C10—H10C	0.9800
			
N2—N1—C1	118.7 (4)	C5—C6—H6	120.0
N2—N1—H1	120.6	C1—C6—H6	120.0
C1—N1—H1	120.6	N2—C7—C8	120.9 (4)
C7—N2—N1	120.8 (4)	N2—C7—Cl2	123.6 (4)
C8—O2—C9	115.0 (4)	C8—C7—Cl2	115.4 (3)
C2—C1—C6	119.7 (4)	O1—C8—O2	125.3 (4)
C2—C1—N1	122.4 (5)	O1—C8—C7	123.1 (4)
C6—C1—N1	117.9 (4)	O2—C8—C7	111.6 (4)
C3—C2—C1	120.2 (5)	O2—C9—C10	106.4 (4)
C3—C2—H2A	119.9	O2—C9—H9A	110.4
C1—C2—H2A	119.9	C10—C9—H9A	110.4
C2—C3—C4	119.8 (5)	O2—C9—H9B	110.4
C2—C3—H3	120.1	C10—C9—H9B	110.4
C4—C3—H3	120.1	H9A—C9—H9B	108.6
C3—C4—C5	120.8 (4)	C9—C10—H10A	109.5
C3—C4—Cl1	120.2 (4)	C9—C10—H10B	109.5
C5—C4—Cl1	119.0 (4)	H10A—C10—H10B	109.5
C6—C5—C4	119.3 (5)	C9—C10—H10C	109.5
C6—C5—H5	120.3	H10A—C10—H10C	109.5
C4—C5—H5	120.3	H10B—C10—H10C	109.5
C5—C6—C1	120.1 (4)		
			
C1—N1—N2—C7	−174.5 (4)	C2—C1—C6—C5	1.2 (7)
N2—N1—C1—C2	−3.4 (7)	N1—C1—C6—C5	−178.3 (4)
N2—N1—C1—C6	176.1 (4)	N1—N2—C7—C8	179.6 (4)
C6—C1—C2—C3	−1.5 (7)	N1—N2—C7—Cl2	2.3 (6)
N1—C1—C2—C3	178.1 (4)	C9—O2—C8—O1	1.1 (6)
C1—C2—C3—C4	0.0 (7)	C9—O2—C8—C7	−178.3 (4)
C2—C3—C4—C5	1.7 (7)	N2—C7—C8—O1	179.5 (4)
C2—C3—C4—Cl1	−178.4 (4)	Cl2—C7—C8—O1	−3.0 (6)
C3—C4—C5—C6	−1.9 (7)	N2—C7—C8—O2	−1.1 (6)
Cl1—C4—C5—C6	178.2 (4)	Cl2—C7—C8—O2	176.4 (3)
C4—C5—C6—C1	0.5 (7)	C8—O2—C9—C10	175.2 (4)

### Hydrogen-bond geometry (Å, °)

**Table:**

*D*—H···*A*	*D*—H	H···*A*	*D*···*A*	*D*—H···*A*
N1—H1···O1^i^	0.86	2.20	3.009 (5)	156

Symmetry codes: (i) −*x*+1, *y*−1/2, −*z*+1.
